# Rethinking treatment strategies for second-degree palmar burns in adults: A ten-year retrospective outcome analysis at a major burn center

**DOI:** 10.1016/j.jpra.2025.06.011

**Published:** 2025-06-30

**Authors:** Martynas Tamulevicius, Florian Bucher, Nadjib Dastagir, Moritz Milewski, Doha Obed, Peter M. Vogt, Khaled Dastagir

**Affiliations:** Department of Plastic, Aesthetic, Hand and Reconstructive Surgery, Hannover Medical School, Carl-Neuberg-Str. 1, d-30625 Hannover, Germany

**Keywords:** Palm burns, Hand burns, Hand function, Burn recovery

## Abstract

**Introduction:**

Hand burns are common, affecting 80 % of burn cases and 40 % of hospitalized patients. Although the palmar surface constitutes only 1.2 % of total body surface area, burns in this region can severely impair hand function. Currently, no standardized treatment guidelines exist. This study analyzes functional and aesthetic outcomes in adult patients with conservatively treated partial-thickness palmar burns.

**Methods:**

A retrospective, single-center cohort study was conducted on adult patients with second-degree palmar burns treated between 2012 and 2022. Functional outcomes were assessed using the Quick Disabilities of the Arm, Shoulder, and Hand (QuickDASH) score, and aesthetic outcomes were evaluated based on skin discoloration, scarring, and patient-reported concerns.

**Results:**

Of 316 cases, 57 (18.0 %) met inclusion criteria. Deep burns were more often treated inpatient (60.0 % vs. 24.4 %, *p* = 0.033) and healed significantly slower (23.87 ± 5.44 vs. 8.41 ± 3.57 days, *p* < 0.001). Functional impairments were comparable between groups (*p* > 0.195), though QuickDASH scores were slightly worse in deep burns (*p* = 0.312). Aesthetic outcomes did not differ significantly. Non-isolated burns were linked to longer healing (*p* < 0.001), worse QuickDASH scores (*p* = 0.015), and more frequent inpatient care (*p* = 0.012).

**Conclusions:**

Deep burns showed significantly longer healing times but no major differences in functional or aesthetic outcomes compared to superficial burns. Non-isolated burns were associated with poorer functional outcomes and longer recovery, suggesting that burn extent impacts healing and rehabilitation needs. These findings support structured follow-up and rehabilitation strategies, emphasizing the need for further research to refine treatment protocols.

## Introduction

Hand burns are among the most common types of burn injuries, accounting for approximately 80 % of cases and 40 % of hospital admissions for burn treatment.^1^ Despite comprising only about 2.4 % of the total body surface area, hand burns can cause significant functional impairments, severely impacting hand function and overall quality of life.^2^, ^3^^–^^5^ Optimal outcomes require a comprehensive treatment approach, including accurate assessment, appropriate wound care, timely surgical intervention when necessary, and intensive physiotherapy.^4^, ^5^, ^6^

Palmar burns are less likely to require surgical intervention than burns in other areas of the body because of the unique properties of the palmar skin, which provide natural resilience and protection.^7^^,^^8^ These protective properties often support a conservative treatment approach, allowing for natural healing and preserving the underlying connective tissue, which is crucial for maintaining grip functionality.^9^ However, in some cases, surgical intervention is unavoidable, particularly for deeper burns or those showing poor healing progress.^9^^,^^10^ Despite the clinical importance of palmar burns, no universally accepted standard exists for determining whether conservative or surgical treatment is more appropriate. Current practices typically involve an initial observation period, with surgical excision and wound coverage performed if necessary.^10^^,^^11^ Additionally, most of the current evidence comes from pediatric populations. In pediatrics, it has been well established that superficial burns heal adequately with conservative treatment, while deeper burns can lead to complications such as scar contractures in the still-developing hand.^12^ Waiting 2–3 weeks to observe the wound healing process before deciding on surgical treatment has proven effective.^13^^,^^14^ However, the lack of a clear consensus on treatment strategies for palmar burns in the adult population underscores the need for further research. This study aims to evaluate the functional and aesthetic outcomes of partial-thickness palmar burns following conservative treatment.

## Methods

### Study design

In this retrospective cohort study, we investigated patients who presented with isolated and non-isolated palmar burns at a hand trauma and replantation center certified by both the Federation of European Societies for Surgery of the Hand and the European Burns Association between January 2012 and December 2022. This analysis included patients transferred to our burn unit, either as primary or secondary referrals. To be included, patients had to be >18 years old and have sustained isolated or non-isolated second-degree palmar burns requiring treatment at the burn center. Burn depth was classified based on standardized clinical criteria routinely used in our department. Superficial partial-thickness burns were characterized by the presence of clear blisters, pink wound beds, brisk capillary refill, and marked pain response to light touch. Deep partial-thickness burns were defined by absent blisters, pale or mottled wound beds, absent capillary refill, and reduced sensitivity. These assessments were performed by at least two experienced burn specialists during initial evaluation.

**Figure 1:**
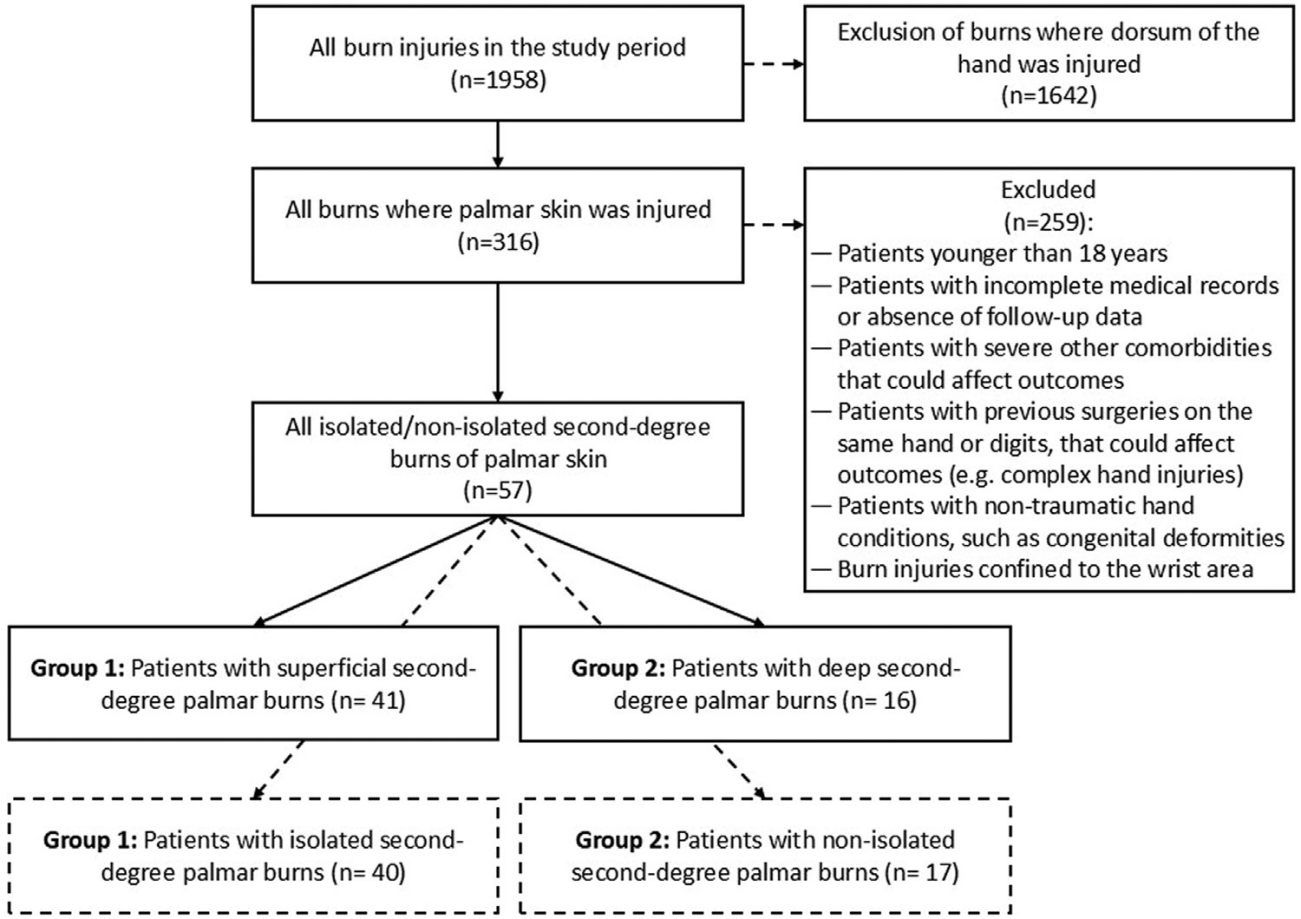
Flow-chart of the study.

All patients were treated conservatively, which was also an inclusion criterion. In our center, conservative management is the standard initial approach for second-degree palmar burns, including selected deep partial-thickness burns, provided there is sufficient perfusion, early signs of epithelialization, and preserved functional positioning. Surgical intervention (e.g., tangential excision and grafting) is considered only if no epithelialization is observed after 2–3 weeks or in the presence of infection or wound deterioration. Patients initially managed conservatively but later requiring surgery were excluded from this analysis to ensure a uniform cohort. Standardized conservative treatment involved daily dressing changes using polyhexanide gel and paraffin gauze after aseptic treatment with a disinfectant (Octenisept; Schülke & Mayr GmbH, Norderstedt, Germany). A sterile gauze was used as a secondary dressing to secure the paraffin gauze, ensuring consistent wound coverage and protection. Additionally, all patients routinely received compression garments, occupational therapy was prescribed, and each patient was scheduled for a follow-up appointment. The exclusion criteria were an age of <18 years, incomplete medical records or missing follow-up data, severe comorbidities that could influence outcomes, prior surgeries on the affected hand or digits, non-traumatic hand conditions (e.g., congenital deformities), and burns confined to the wrist area (Figure 1).

Data from all patients admitted to and treated in the department’s burn care unit were retrospectively collected through an online hospital database. In cases where follow-up visit records were incomplete, patients were contacted by phone to retrieve the missing data. All data were collected and stored anonymously. Because this was a retrospective analysis of an anonymous database utilizing routine data, the requirement for approval from the local ethics committee was waived.

Functional outcomes were assessed using the following methods. Complete finger flexion and extension ability was measured by evaluating the range of motion in the affected hand. Subjective sensitivity was assessed through patient self-reporting, differentiating between no sensation, reduced sensation (with at least protective sensation preserved), and normal sensation. The Quick Disabilities of the Arm, Shoulder, and Hand (QuickDASH) questionnaire was completed to evaluate physical function and symptoms.^15^ Aesthetic outcomes were evaluated based on the presence of visible skin discoloration or scarring resulting in skin tension at the injury site. Although validated scar assessment tools such as the Vancouver Scar Scale (VSS) are recognized, they are not routinely used in our department unless the scar is deemed clinically significant or a specific scar therapy requires follow-up evaluation. Instead, aesthetic assessments are conducted by at least two burn specialists and documented descriptively. For the purposes of this study, we retrospectively aligned available clinical documentation with selected VSS-based criteria to enhance consistency and comparability. Scars were classified as “significant” if they met at least two of the following thresholds: vascularity ≥2 (red or purple), pigmentation ≥1 (hypo- or hyperpigmentation), pliability ≥2 (yielding or worse), and height ≥2 (≥2 mm). In routine clinical practice, formal scar scales are not applied if the scar is considered aesthetically and functionally insignificant.

### Statistical analysis

Metric data are presented as mean ± standard deviation, with minimum and maximum values provided. Ordinal data are shown as counts and percentages. All data were tested for a normal distribution. Statistical analyses were performed using SPSS (IBM Corp., Armonk, NY, USA) and GraphPad Prism version 9.5.1 (GraphPad Software, San Diego, CA, USA). Statistical significance was defined as *p* < 0.05. Initial comparisons between groups were performed Pearson’s chi-square test for nominal data, and Fisher’s exact test was applied if more than 20 % of expected frequencies were below 5. For pairwise comparisons of normally distributed metric data, the unpaired *t*-test was used. The Mann–Whitney U test was used for non-normally distributed metric data between two groups.

## Results

### Patient demographics

**Figure 2:**
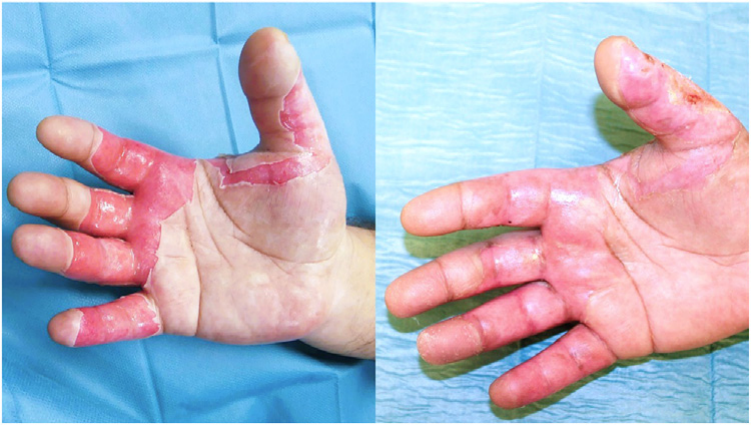
A 43-year-old patient with superficial second-degree palmar burns at presentation after initial blister debridement (left) and at follow-up 7 days post-injury with fully reepithelialized palmar skin (right).

Of 1958 burn cases treated during the study period, 316 (16.1 %) involved palmar skin injuries. After applying the inclusion and exclusion criteria, 57 patients (2.9 % of all burn cases, 18.0 % of those with palmar burns) met the study criteria and were included in the analysis (Figure 1). Of these, 41 patients (71.9 %) had superficial second-degree burns (Figure 2), while 16 patients (28.1 %) had deep second-degree burns. Most patients were male (71.9 %), with no significant difference between burn severity groups (*p* = 0.740). The mean age of the cohort was 40.33 ± 14.79 years, with no statistically significant difference between patients with superficial and deep burns (*p* = 0.389). Similarly, the body mass index was comparable between groups (24.37 ± 4.67 vs. 25.62 ± 4.12 kg/m^2^, *p* = 0.497). The smoking prevalence was slightly higher in the deep second-degree burn group (31.6 %) than in the superficial burn group (21.9 %), but this difference was not statistically significant (*p* = 0.493). The prevalence of diabetes (12.3 %) and peripheral arterial disease (7.0 %) was low across the cohort, with no significant differences between groups (*p* = 0.978 and *p* = 0.985, respectively).

Most patients (86.0 %) were right-handed, with no significant difference between groups (*p* = 0.673). Isolated hand burns occurred in 70.2 % of cases, while 26.3 % of patients had bilateral hand burns, with no statistically significant difference between groups (*p* = 0.434 and *p* = 0.498, respectively). Burn localization was also similar, with 45.6 % of patients having isolated palm burns, 15.8 % having isolated palmar finger burns, and 38.6 % having burns affecting both palm and fingers (*p* > 0.660 for all comparisons).

Regarding burn etiology, flame burns were the most common cause (49.1 %), followed by contact burns (26.3 %), fat burns (13.3 %), scalds (7.0 %), and chemical burns (5.3 %). There were no statistically significant differences in etiology between burn severity groups (*p* > 0.190 for all comparisons), although flame burns appeared more common in the deep second-degree burn group (62.5 %) than in the superficial burn group (43.9 %). Chemical burns were only present in the superficial burn group, but the small sample size limits interpretation (*p* = 0.552). Overall, no statistically significant differences were found between patients with superficial and deep second-degree burns in terms of demographics, burn characteristics, or etiology. The relatively small sample size in some subcategories may have limited the ability to detect potential differences. Patient and burn characteristics are summarized in Table 1.

**Table 1:** Patient demographics and characteristics.

	All (*n* = 57)	Superficial second-degree burns (*n* = 41)	Deep second-degree burns (*n* = 16)	*p* value
Sex (male)	41 (71.9 %)	30 (73.2 %)	11 (73.3 %)	0.740
Age (years)	40.33 ± 14.79	39.27 ± 15.46	43.06 ± 12.9	0.389
Body mass index (kg/m^2^)	24.98 ± 5.47	24.37 ± 4.67	25.62 ± 4.12	0.497
Smoker	14 (24.6 %)	9 (21.9 %)	5 (31.6 %)	0.493
Diabetes	7 (12.3 %)	5 (12.2 %)	2 (12.5 %)	0.978
Peripheral arterial disease	4 (7.02 %)	3 (7.32 %)	1 (6.25 %)	0.985
Handedness (right-handed)	49 (86.0 %)	36 (87.8 %)	13 (81.3 %)	0.673
Isolated hand burns	40 (70.2 %)	30 (73.2 %)	10 (62.5 %)	0.434
Bilateral hand burns	15 (26.3 %)	10 (24.4 %)	5 (31.3 %)	0.498
Burned area of palmar hand				
Palmar finger(s)	9 (15.8 %)	7 (17.1 %)	2 (12.5 %)	0.664
Palm of hand	26 (45.6 %)	19 (46.3 %)	7 (43.8 %)	0.860
Palm and palmar finger(s)	22 (38.6 %)	15 (36.6 %)	7 (43.8 %)	0.763
Etiology				
Flame	28 (49.1 %)	18 (43.9 %)	10 (62.5 %)	0.242
Contact burns	15 (26.3 %)	13 (31.7 %)	2 (12.5 %)	0.190
Fat burns	7 (13.3 %)	5 (12.2 %)	2 (12.5 %)	1.000
Scalds	4 (7.0 %)	2 (4.9 %)	2 (12.5 %)	0.312
Chemical burns	3 (5.3 %)	3 (7.3 %)	0 (0.0 %)	0.552
Burned body surface area (%)	3.62 ± 6.81	3.37 ± 7.66	4.25 ± 3.97	0.668
ABSI^a^	4.05 ± 0.92	4.02 ± 0.88	4.13 ± 1.03	0.713

Data are presented as *n* (%) or mean ± standard deviation.

^a^ASBI, abbreviated burn severity index.

### Outcomes

The mean follow-up time was 52.7 ± 23.9 months, with no significant difference between burn types (*p* = 0.382). One-third of patients required inpatient treatment, more frequently in non-isolated burns (70.6 % vs. 17.5 %, *p* < 0.001). The mean hospital stay was 4.67 ± 3.17 days, with no significant difference between groups (*p* = 0.067). The time to full reepithelialization was significantly longer in non-isolated burns for superficial second-degree burns (10.73 ± 6.42 vs. 7.55 ± 0.77 days, *p* = 0.009), whereas no significant difference was found for deep second-degree burns (26.43 ± 2.37 vs. 22.56 ± 6.67 days, *p* = 0.167). Functional limitations were observed in 14.0 % of cases, with no significant group difference. Reduced skin sensitivity affected 8.8 % of patients. The mean QuickDASH score was 10.75 ± 6.52, significantly higher in non-isolated burns (14.88 ± 7.07 vs. 9.00 ± 5.47, *p* = 0.001). Rehabilitation measures were similar between groups, with no significant differences in hand therapy (*p* = 0.583) or compression garment use (*p* = 0.583 and *p* = 0.767, respectively). The mean burned body surface area was significantly larger in non-isolated burns (11.67 % ± 2.83 % vs. 1.89 % ± 0.29 %, *p* = 0.013). The mean ABSI score was 4.05 ± 0.92, with a slightly higher ABSI score in deep second-degree burns. However, no significant difference between groups could be identified (*p* = 0.107). Aesthetic outcomes did not significantly differ between superficial and deep second-degree burns. No patient required surgical therapy or scarring treatment. There was no occurrence of keloid formation. None of the patients lost sensory function of the palmar hand. The patients’ outcomes are summarized in Table 2.

**Table 2:** Surgical and functional outcomes for superficial and deep second-degree palmar burns.

	All (*n* = 57)	Superficial second-degree burns (*n* = 41)	Deep second-degree burns (*n* = 16)	*p* value
Follow-up time (months)	52.7 ± 23.9	56.8 ± 23.6	48.5 ± 24.2	0.382
Inpatient treatment	19 (33.3 %)	10 (24.4 %)	9 (60.0 %)	**0.033**
Length of stay (days)	4.67 ± 3.17	3.92 ± 2.42	5.67 ± 3.87	0.219
Time to full reepithelialization of burned skin (days)	12.46 ± 7.99	8.41 ± 3.57	23.87 ± 5.44	**<0.001**
Incomplete finger flexion and/or extension ability	8 (14.0 %)	6 (14.3 %)	2 (13.3 %)	1.000
Reduced sensitivity of burned skin areas	5 (8.8 %)	4 (9.8 %)	1 (6.3 %)	0.432
QuickDASH score	10.75 ± 6.52	10.12 ± 6.44	12.13 ± 6.81	0.312
Hand therapy	14 (24.6 %)	5 (12.2 %)	9 (60.0 %)	**0.002**
Compression garments	12 (21.1 %)	0 (0.0 %)	12 (80.0 %)	**<0.001**
Aesthetic outcomes				
Skin discoloration	11 (19.3 %)	8 (19.5 %)	3 (18.8 %)	1.000
Significant scarring	10 (17.5 %)	7 (17.1 %)	3 (18.8 %)	0.966
No aesthetic concerns	42 (73.7 %)	30 (73.2 %)	12 (75.0 %)	1.000

Data are presented as *n* (%) or mean ± standard deviation.

Significance of bold values statistically significant if p < 0.05.

### Outcomes of isolated vs. non-isolated palmar burns

Among 57 patients, non-isolated palmar burns were more frequently deep (41.2 % vs. 22.5 %, *p* = 0.159) and required inpatient treatment significantly more often (70.6 % vs. 17.5 %, *p* < 0.001). The duration of hospital stay was longer in the non-isolated group (5.54 ± 3.55 vs. 3.25 ± 1.83 days, *p* = 0.067). Reepithelialization of the affected hand for superficial second-degree burns took significantly longer in non-isolated burns (10.73 ± 6.42 vs. 7.55 ± 0.77 days, *p* = 0.009), while deep second-degree burns healed at a similar rate (26.43 ± 2.37 vs. 22.56 ± 6.67 days, *p* = 0.167). Functional impairments, including incomplete finger flexion and/or extension (14.0 %) and reduced sensitivity (8.8 %), were more common in non-isolated burns (23.5 % and 17.6 %, respectively), but the differences did not reach statistical significance (*p* = 0.195 and *p* = 0.142). The mean QuickDASH score was significantly worse in non-isolated burns (14.88 ± 7.07 vs. 9.00 ± 5.47, *p* = 0.001). Rehabilitation measures, including hand therapy (*p* = 0.583) and compression garment use (*p* = 0.767), were comparable between groups. Non-isolated burns were associated with a significantly larger burned body surface area (11.67 % ± 2.83 % vs. 1.89 % ± 0.29 %, *p* = 0.013). The mean ABSI score was slightly higher in non-isolated burns (4.35 ± 0.99 vs. 3.93 ± 0.86), but the difference was not statistically significant (*p* = 0.107). Skin discoloration was more frequent in non-isolated burns (35.3 % vs. 12.5 %), but this difference was not statistically significant, suggesting that the burn extent may influence pigmentation changes without a clear impact on overall aesthetic outcomes. There was no occurrence of keloid formation. Outcomes of isolated and non-isolated palmar burns are summarized in Table 3.

**Table 3:** Surgical and functional outcomes for isolated and non-isolated palmar burns.

	All (*n* = 57)	Isolated (*n* = 40)	Non-isolated (*n* = 17)	*p* value
Deep second-degree burns	16 (28.1 %)	9 (22.5 %)	7 (41.2 %)	0.159
Inpatient treatment	19 (33.3 %)	7 (17.5 %)	12 (70.6 %)	**<0.001**
Length of stay (days)	4.67 ± 3.17	3.25 ± 1.83	5.54 ± 3.55	0.067
Time to full reepithelialization (days)				
Superficial second-degree burns	8.41 ± 3.57	7.55 ± 0.77	10.73 ± 6.42	**0.009**
Deep second-degree burns	23.87 ± 5.44	22.56 ± 6.67	26.43 ± 2.37	0.167
Incomplete finger flexion and/or extension ability	8 (14.0 %)	4 (10.0 %)	4 (23.5 %)	0.195
Reduced sensitivity of burned skin areas	5 (8.8 %)	2 (5.0 %)	3 (17.6 %)	0.142
QuickDASH score	10.75 ± 6.52	9.00 ± 5.47	14.88 ± 7.07	**0.001**
Hand therapy	14 (24.6 %)	9 (22.5 %)	5 (29.4 %)	0.583
Compression garments	12 (21.1 %)	8 (20.0 %)	4 (23.5 %)	0.767
Burned body surface area (%)	3.62 ± 6.81	1.89 ± 0.29	11.67 ± 2.83	**0.013**
ABSI^a^	4.05 ± 0.92	3.93 ± 0.86	4.35 ± 0.99	0.107
Aesthetic outcomes				
Skin discoloration	11 (19.3 %)	5 (12.5 %)	6 (35.3 %)	0.103
Significant scarring	10 (17.5 %)	8 (20.0 %)	2 (11.8 %)	0.706
No aesthetic concerns	42 (73.7 %)	31 (77.5 %)	11 (64.7 %)	0.500

Data are presented as *n* (%) or mean ± standard deviation.

^a^ABSI, abbreviated burn severity index.

Significance of bold values statistically significant if p < 0.05.

## Discussion

Compared with other body regions, palmar skin exhibits specialized anatomical and physiological features, including thickened layers, distinct cellular composition, and enhanced mechanical and regulatory capabilities, which make it much less vulnerable to injuries such as burns. It has a thicker epidermis, with additional layers in the stratum spinosum and granulosum.^16^ The stratum corneum in palmar skin shows nearly sevenfold greater intercellular cohesion than in hairy skin.^17^ Vela-Romera et al.^8^ found that palmar skin has a higher number of epithelial layers and larger cell sizes in most strata compared with non-ridged skin. Unique ridge patterns and a cellular composition marked by an abundance of Merkel cells (with fewer melanocytes and Langerhans cells) further distinguish its tactile and barrier properties.^8^ At the dermal–epidermal junction, studies document a complex basement membrane topography, with grooves and papillae that support increased numbers of spindle-shaped cells and specialized capillary structures.^18^ This finding suggests unique vascular arrangements at the dermo-epidermal junction of palmar skin, which might contribute to better healing properties. Functional findings include refined moisture regulation through sweat pore activity, enhanced friction and mechanical stress resistance for improved grip, and dynamic vascular control that also buffers blood pressure changes. Together, these features delineate the unique structural and physiological adaptations of palmar skin. Currently, there is a lack of direct comparative studies examining healing times between palmar skin and hairy skin. The palm’s robust blood supply contributes not just to rapid healing, but also potentially to healing without scarring or pigmentation disturbances. Our study supports this hypothesis because we found no significant differences in functional and aesthetic outcomes between superficial and deep second-degree burns. Despite deeper burns requiring inpatient treatment more frequently, functional impairments remained comparable between groups (*p* > 0.195), and most patients reported no long-term aesthetic concerns. These findings highlight the palm’s ability to recover well, even in deeper burns, possibly because of its distinct basement membrane structure and efficient vascularization at the dermo-epidermal junction.

Current management of palmar burns primarily involves an initial observation period, with surgical intervention reserved for cases where healing is unlikely with conservative treatment.^10^^,^^11^ This approach is well-established in pediatric populations, in which superficial burns heal conservatively, while deeper burns may lead to contractures if not managed appropriately.^19^ In children, waiting 2–3 weeks before considering surgical treatment has been a widely accepted strategy.^12^ However, evidence for adult palmar burns remains limited. Studies in children have shown that 87 % of pediatric palmar burns heal conservatively.^19^ However, in children, delayed healing beyond 21 days is associated with a 78 %–100 % risk of hypertrophic scarring, which supports a watchful waiting approach before considering excision and grafting.^12^ By contrast, our study specifically measured time to full reepithelialization rather than complete healing, which explains why our reported healing times appear shorter than those in pediatric studies. Superficial burns in adults reepithelialized in 8.41 days, which aligns with early stages of healing in pediatric cases. However, deep burns took an average of 23.87 days to reepithelialize, exceeding the 21-day threshold used in pediatric studies to determine the possible need for surgical intervention.

Previous studies have shown that delayed wound closure can significantly increase the risk of hypertrophic scarring and keloid formation.^20^^,^^21^ However, in our patient cohort, only a few cases of significant hypertrophic scarring were observed, and no keloids developed. Despite the prolonged healing duration, none of our patients required surgery, suggesting that the reepithelialization time alone may not be a sufficient criterion for surgical decision-making in adults. Instead, monitoring for functional deficits and scarring potential may be more relevant in guiding treatment choices. While previous studies suggest that early excision and grafting may accelerate healing, our findings provide strong evidence supporting conservative management of palmar burns, demonstrating that long-term functional and aesthetic outcomes were favorable even without surgical intervention.^22^

Histological and molecular studies have shown that deep partial-thickness burns are characterized by increased apoptotic activity, not only in the dermis but also in the cutaneous adnexa, unlike superficial or even full-thickness burns.^23^ This apoptosis is often driven by ischemic damage, where inadequate perfusion forces otherwise viable cells into programmed cell death. However, the palmar region, which is rich in vascular and nerve supply and generally protected by thicker epidermis and a denser microvascular network, may offer a more favorable microenvironment for spontaneous healing even in deep partial burns. This is particularly relevant in adult patients, where delayed epithelialization does not always correlate with poor outcomes. Unlike pediatric populations, older adults show altered wound healing physiology, including reduced vascular perfusion, dermal thinning, and a less robust inflammatory response. These changes slow reepithelialization but may also reduce excessive fibroblast activity and scarring risk. Romanowski et al.^24^ highlighted how aging skin, with fewer dermal papillae and reduced keratinocyte turnover, heals more slowly but not necessarily with worse functional or aesthetic outcomes. Furthermore, a randomized controlled trial by Zacharevskij et al.^25^ compared non-surgical treatments for deep partial-thickness hand burns and demonstrated that hydrocolloid dressings, also employed in our cohort, significantly improved both healing time and scar quality. Scar severity, assessed using the VSS, was very low in the hydrocolloid group (mean VSS = 1.36). In our clinical setting, a VSS below 2 in all domains is generally considered aesthetically and functionally insignificant and would not typically trigger scar-specific interventions. Notably, these results were observed in burns with preserved perfusion, as confirmed by Laser Doppler Imaging (260–600 perfusion units), emphasizing that tissue perfusion rather than burn depth alone may be a more accurate predictor of spontaneous healing potential in the palm.^25^

The QuickDASH normative data indicate that average scores vary with age, with younger adults (20–29 years) having mean scores of 5–6, while scores increase with age, reaching 22–36 in those over 70 years old.^26^ In our study, the mean QuickDASH score was 10.75 ± 6.52, which is slightly higher than the general population norms for younger adults but lower than expected for older individuals. Given that our cohort had an average age of 40.33 years, our QuickDASH scores are comparable to or slightly above normative values for this age group. This suggests that despite the functional challenges associated with palmar burns, long-term disability is generally mild, supporting the effectiveness of conservative treatment in preserving hand function. These findings align with Sheridan et al.^22^’s results in pediatric populations, indicating that although early excision shortens the healing time, it does not necessarily lead to improved long-term functional outcomes.^27^ Given that functional impairments in our study were minimal and aesthetic concerns were rare, conservative treatment appears justified for most palmar burns in adults, particularly superficial and select deep burns.

Palmar burns can occur as isolated injuries or in combination with burns affecting other body regions. Our study showed that non-isolated palmar burns resulted in longer healing times, worse QuickDASH scores, and higher rates of inpatient treatment. This suggests that the overall burn burden, rather than just the depth of the palmar injury, plays a critical role in recovery. Possible contributing factors include increased systemic inflammatory responses, prolonged immobilization, or compromised perfusion in patients with more extensive burns. Despite these differences in functional recovery, aesthetic outcomes were not significantly affected by burn extent or severity (*p* = 0.500). This supports the idea that the unique anatomical properties of palmar skin contribute to favorable scarring outcomes, even in patients with larger burns. Findings from previous research align with our results. Studies comparing isolated and non-isolated hand burns found that non-isolated burns were more likely to require hospitalization (54 % vs. 6 %) and be associated with full-thickness injuries.^28^ This highlights the increased severity and resource utilization in non-isolated cases, necessitating more structured rehabilitation and inpatient care. Additionally, non-isolated burns often involved bilateral hand injuries, which prolong functional recovery and may limit return to work.^28^ In our study, patients with non-isolated palmar burns had significantly worse QuickDASH scores (14.88 vs. 9.00, *p* = 0.001), reinforcing the impact of total burn burden on hand function. Non-isolated palmar burns pose greater challenges in functional recovery than do isolated injuries, emphasizing the importance of early rehabilitation and inpatient care for more extensive burns. However, the resilience of palmar skin allows good aesthetic outcomes, even in severe cases. These findings support a conservative-first approach for most palmar burns while stressing the need for individualized rehabilitation protocols to address functional impairments in non-isolated injuries.

This study has several limitations. First, as a retrospective, single-center analysis, its findings may not be generalizable to other institutions with differing treatment protocols or patient populations. The relatively small sample size, despite complete follow-up, may have limited the statistical power to detect smaller differences between groups. Selection bias is possible, as only patients with completed conservative treatment and adequate documentation were included, potentially excluding more severe or surgically treated cases.

Another limitation is the absence of standardized objective functional assessments, as outcomes were primarily based on clinical documentation and patient-reported measures. Although the QuickDASH questionnaire is a validated and appropriate tool for assessing upper limb function, it does not capture objective parameters such as grip strength, range of motion, or dexterity. These assessments were not consistently documented in the retrospective dataset and therefore could not be analyzed. Future prospective studies should incorporate such objective measures to provide a more comprehensive evaluation of hand function after burn injuries. Although variability in follow-up intervals and clinical note detail is inherent to retrospective designs, reepithelialization time was carefully reconstructed from available records to reflect real-world healing dynamics. Burn depth classification followed standardized criteria and was consistently assessed by at least two experienced burn specialists, minimizing inter-observer variability. However, the lack of objective tools such as laser Doppler imaging or histopathology still limits external reproducibility. Wound infections were inconsistently documented and therefore excluded, possibly underestimating their effect on healing. Lastly, because the study focused exclusively on conservatively treated cases, no conclusions can be drawn about surgical outcomes. Future multicenter prospective studies with standardized diagnostics are needed to refine treatment strategies for palmar burns.

## Conclusions

Palmar burns, although small in surface area, can significantly impact hand function and recovery. Our findings confirm the effectiveness of conservative treatment, even in deep burns, while highlighting the challenges of non-isolated injuries, which prolong healing and worsen functional outcomes. This study emphasizes the importance of early assessment, structured rehabilitation, and individualized treatment. Superficial burns heal well with conservative care and early mobilization, whereas deep and non-isolated burns require closer monitoring and targeted rehabilitation. Although conservative treatment remains effective, tailored follow-up is essential to prevent long-term deficits. The lack of clear adult guidelines calls for further research to refine treatment protocols and surgical indications to optimize recovery.

## Authors' contributions

MT collected and analyzed data, wrote the draft and final version of the manuscript. KD had the project idea, mentored the study, and revised the manuscript. FB, ND, MM und DO revised the manuscript. PMV mentored the study, revised the manuscript. All authors reviewed and approved the final manuscript.

## Data availability statement

The data supporting the findings of this study are available from the corresponding author upon reasonable request. Due to privacy concerns, the dataset is not publicly archived. Access to the data will be provided in compliance with institutional guidelines and applicable regulations to ensure individual privacy is protected.

## Funding

The authors received no financial support for the research, authorship, and/or publication of this article.

## Ethical approval declaration

Ethical approval was waived by the local Ethics Committee in view of the retrospective nature of the study and all the procedures being performed were part of routine care. The study was performed in accordance with the ethical standards of the institutional and national research committee and with the 1964 Helsinki declaration and its later amendments.

## Informed consent declaration

Informed consent was not obtained as a study was done in an anonymized retrospective manner.

## Declaration of competing interest

All authors declare that they have no conflicting interests.
